# Computational assessment of the effects of a pulsatile pump on toxin removal in blood purification

**DOI:** 10.1186/1475-925X-9-31

**Published:** 2010-06-22

**Authors:** Ki Moo Lim, Eun Bo Shim

**Affiliations:** 1 Department of Mechanical & Biomedical Engineering, Kangwon National University, Chuncheon, Kangwon-do 200-701, Republic of Korea

## Abstract

**Background:**

For blood purification systems using a semipermeable membrane, the convective mass transfer by ultrafiltration plays an important role in toxin removal. The increase in the ultrafiltration rate can improve the toxin removal efficiency of the device, ultimately reducing treatment time and cost. In this study, we assessed the effects of pulsatile flow on the efficiency of the convective toxin removal in blood purification systems using theoretical methods.

**Methods:**

We devised a new mathematical lumped model to assess the toxin removal efficiency of blood purification systems in patients, integrating the mass transfer model for a human body with a dialyser. The human body model consists of a three-compartment model of body fluid dynamics and a two-compartment model of body solute kinetics. We simulated three types of blood purification therapy with the model, hemofiltration, hemodiafiltration, and high-flux dialysis, and compared the simulation results in terms of toxin (urea and beta-2 microglobulin) clearance and the treatment dose delivered under conditions of pulsatile and non-pulsatile pumping. *In vivo *experiments were also performed to verify the model results.

**Results:**

Simulation results revealed that pulsatile flow improved the convective clearance of the dialyser and delivered treatment dose for all three types of therapy. Compared with the non-pulsatile pumping method, the increases in the clearance of urea and beta-2 microglobulin with pulsatile pumping were highest with hemofiltration treatment (122.7% and 122.7%, respectively), followed by hemodiafiltration (3.6% and 8.3%, respectively), and high-flux dialysis (1.9% and 4.7%, respectively). EKRc and std Kt/V averaged 28% and 23% higher, respectively, in the pulsatile group than in the non-pulsatile group with hemofiltration treatment.

**Conclusions:**

The pulsatile effect was highly advantageous for all of the toxins in the hemofiltration treatment and for β_2_-microglobulin in the hemodiafiltration and high-flux dialysis treatments.

## Background

Blood purification systems employing semipermeable membranes are widely used in the treatment of patients with chronic renal failure (CRF). The mechanisms underlying toxin removal in these systems are diffusion and convection [1,2]. As shown in Table 1, the convective mass transfer by ultrafiltration (UF) plays an important role in toxin removal [2]. Thus, the increase in the UF rate can improve the toxin removal efficiency of the device, eventually requiring less treatment time and cost.

**Table 1:** Blood purification systems using semipermeable membranes

Type of blood purification system	Mechanism of toxin removal
Conventional hemodialysis	Dialysis
HFx	Dialysis + ultrafiltration
HF	Ultrafiltration
HDF	Dialysis + ultrafiltration

Several experimental studies have indicated that the UF rate in blood purification systems can be increased by using a pulsatile pump instead of a non-pulsatile pump [3,4]. More specifically, our previous study demonstrated that pulsatile flow can generate a greater UF rate than non-pulsatile flow due to the increased transmembrane pressure (TMP) and water permeability of the membrane [3]. However, these studies were limited to basic *in vitro *experiments to observe the UF efficiency of the dialyser device itself. To our knowledge, *in vivo *evaluations of the device and its application to patients have not been performed due to difficulties in developing an appropriate animal model. Although animal experiments regarding urea removal during blood purification treatment are relatively easy to perform, serious difficulties exist in establishing an *in vivo *animal model with a high ྞ _2_-microglobulin (B2M) concentration. As noted previously by Cameron [5], B2M, which is another significant toxin in blood purification, accumulates slowly in the body under CRF conditions, and therefore, a great deal of time is required for renal failure associated with a high B2M concentration. However, *in vivo *analysis is inevitable for a reasonable assessment of the toxin removal efficiency.

Here, we use a theoretical method as an alternative to *in vivo *experiment to assess the effects of pulsatile pump on toxin removal. For this purpose, we devised a new mathematical model to assess the toxin removal efficiency of blood purification systems in patients, integrating the mass transfer model for a human body [6] with a dialyser in a blood purification system [2]. The present study focused on whether pulsatile flow can improve the delivered treatment dose for patients during blood purification. We performed a simple *in vivo *clinical test to verify the present simulation model by comparing the simulated urea concentration profile with clinical observations.

Using the mathematical method, we then predicted the dialyser clearances of urea and B2M for various blood purification therapies using pulsatile and non-pulsatile systems. In addition, the treatment dose delivered to patients with CRF was predicted under conditions of pulsatile and non-pulsatile blood purification therapy with a long-term duration of 5 weeks using common blood purification systems, such as hemofiltration (HF), hemodiafiltration (HDF) and high-flux dialysis (HFx).

## Methods

To compare toxin removal efficacy between pulsatile and non-pulsatile systems for the three types of blood purification therapy (*i.e.*, HF, HDF and HFx), we used a computational model with *in vitro *and *in vivo *experiments. The *in vitro *experiment to analyze the contribution of pulsatile flow to the UF rate has already been reported [3] and is summarized in Appendix A1. We identified parameters used in the computational model from the results of the *in vitro *experiment, such as the pressures of the dialyser inlet and outlet of the blood circuit, and the UF coefficient (*K*_*uf*_) of the dialyser according to the blood pumping rate (*Q*_*b*_) and pumping type (pulsatile *vs*. non-pulsatile). From these parameters, we theoretically calculated the convective clearance for each type of therapy. By inserting the calculated convective clearance into the computational model, we can simulate the hemodynamic and physiological conditions of hemodialysis patients and assess the effects of pump type and pulsatility on toxin removal efficacy of the patients with a long-term duration. To validate the present computational model, the computation results of urea removal in patients with CRF were compared with the results observed *in vivo*.

### Computational model of the diffusion and convection in the dialyser system

The mass transfer inside the body that occurs during blood purification therapy can be explained roughly using body solutes and fluid exchange kinetics, as described by Ursino *et al*. [6]. To simulate the effect of pulsatile flow in a dialyser on the convective clearance of the three types of blood purification therapy, *i.e.*, HF, HDF, and HFx, we modified the dialyser model of Depner and Garred [2] for each therapy and combined this with the human body model of Ursino *et al*. The human body model is explained briefly below, and the dialyser model for each therapy is explained in the following two sections.

**Figure 1:**
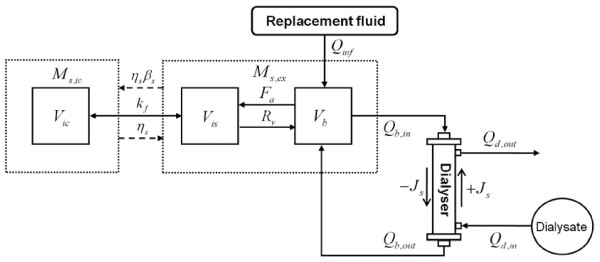
**Schematic diagram of the mathematical model for a patient with the blood purification system**. There are two compartments for solute kinetics and three compartments for fluid dynamics inside body. (------), solute transfer; (----), fluid transfer; *ic*, intracellular; *is*, interstitial; *pl*, plasma;*ex*, extracellular; *b*, blood; *d*, dialysate; *η_s_*, mass transfer coefficient of solute *s*between the intracellular and extracellular compartments; *β_s_*, equilibrium ratio of solute *s; V*, compartment volume; *M_s_*, mass of the solute *s; Q_inf_*, flow rate of replacement fluid; *J_s_*, removal rate of solute *s *across the dialyser; *R_v_*, fluid reabsorption rate at the venous capillaries; *F_a_*, fluid filtration rate at the arterial capillaries; *k_f_*, osmotic filtration coefficient at the cellular membrane; *Q*, extracorporeal flow rate; *in*, inlet; *out*, outlet. Modified from Ursino *et al*. [6] and Depner and Garred [2].

Body fluid consists of three compartments (intracellular, interstitial and plasma) in the model, and two compartments (intracellular and extracellular) are used to formulate the kinetics of the solutes. Fluid exchange between the intracellular and interstitial compartments results from their osmotic pressure difference, whereas oncotic and hydrostatic pressure gradients induce fluid transfer between the interstitial fluid and plasma. Fluid exchange between the plasma and dialysate is determined by the UF rate, which is the product of transmembrane pressure through the dialyser membrane and UF coefficient of the dialyser. Diffusion is the main mechanism of solute movement into and out of cells. In the extracellular compartment, the solute kinetics is influenced by mass transfer through the dialyser in a blood purification system. Here, the model variables related to solutes include urea and B2M as markers of small and large molecular toxins, respectively, and other important electrolytes (sodium, potassium and chloride) and proteins. As the meaningful variables were urea and B2M, we discuss only the variations in urea and B2M, which are markers of uraemic toxins. A schematic diagram of the present mathematical model is shown in Figure 1. A detailed explanation of the governing equations and parameters adopted from Ursino *et al*. [6] is presented in Appendix A2 and Table 2, respectively.

**Table 2:** Parameter assignment of the mathematical model

	Definition	Value	Units	Ref.
*KoA*_*urea*_	Diffusive mass transfer coefficient of the dialyser (urea)	967	mL/min	Specs.
*KoA*_*B2M*_	Diffusive mass transfer coefficient of the dialyser (B2M)	290	mL/min	Specs.
*Si*_*urea*_	Dialyser sieving coefficient (urea)	1		Specs.
*Si*_*B2M*_	Dialyser sieving coefficient (B2M)	0.8		Specs.
*η*_*urea*_	Transfer coefficient (urea)	0.77	L/min	6
*η*_*B2M*_	Transfer coefficient (B2M)	0.077	L/min	2
*k*_*f*_	Water-transfer coefficient	0.24	L^2^/min/mmol	6
*β*_*urea*_	Equilibrium ratio (urea)	1		6
*β*_*B2M*_	Equilibrium ratio (B2M)	1		Est.
*R*	Plasma water fraction	0.94		6
*G*	Urea generation rate	6.24	mg/min	2

More detailed cellular and vascular parameters were described previously by Ursino *et al*. [6]. Est., estimated; Specs., from the FX60 dialyser specifications.

### Convective toxin clearance for HF, HDF and HFx treatments

HF therapy works by passing the patient's blood through a dialyser that filters out waste products and water and then adds replacement fluid before returning the blood to the body. The replacement fluid maintains fluid volume in the blood and provides electrolytes. The only mechanism of toxin removal in HF therapy is convective transport through the dialyser membrane. HDF is a method that combines hemofiltration and counter-current dialysis for diffusive transport. The dialysate flows in the direction opposite the blood flow in order to maintain the maximum concentration gradient across the membrane, and the counter-current flow condition increases the efficiency of dialysis. Consequently, HDF uses both replacement fluid and dialysate solution, and the mechanism of toxin removal in HDF is both convective and diffusive transport. HFx therapy also provides both convective and diffusive transport simultaneously, but it uses only dialysate solution, not replacement fluid. The pressure distribution along the length of the dialyser in both the blood and dialysate compartment is an important factor in convective transport. At the dialyser inlet of the blood circuit is a region of filtration, as the blood pressure exceeds the dialysate pressure. Backfiltration occurs at the region of the dialyser outlet of the blood circuit, as the dialysate pressure exceeds the blood pressure.

Convective toxin clearance is a direct input parameter for the present simulation code and is computed separately for each blood purification therapy by computing the UF rate. The UF rate can be calculated as the product of the mean transmembrane pressure (TMPm) and UF coefficient, shown in the following equations:(1)(2)

where *P*_*b,in *_and *P*_*b,out *_are the mean pressures at the dialyser inlet and outlet of the blood circuit, respectively, *P*_*d,in *_and *P*_*d,out *_are those of the dialysate circuit, *K*_*uf *_is the UF coefficient of the dialyser and *Q*_*f *_is the UF rate.

For the HF treatment, *P*_*d,in *_and *P*_*d,out *_were set to zero and *P*_*b,in *_and *P*_*b,out *_were obtained from the *in vitro *reference data. Based on these pressures, TMPm was computed using Eq. (1). We calculated the UF rate (*Q*_*f*_) in Eq. (2) from the UF coefficient obtained from the *in vitro *experiment and the TMPm. Then, the convective toxin clearance for the HF treatment was calculated with Eq. (A11) shown in the Appendix using the UF rate.

In the HDF treatment, both diffusive and convective clearances exist simultaneously. We set the dialysate flow rate to 300 mL/min as in the *in vitro *experiments. Diffusive toxin clearance for the HDF treatment can be computed from Eq. (A8) in the Appendix using the specified values of the blood and dialysate flow rate through the dialyser. Based on the pressures and UF coefficients obtained from the *in vitro *experiments, UF rate was calculated using Eq. (1) and (2). Then, convective toxin clearance for the HDF treatment was calculated using Eqs. (A9) and (A10) shown in the Appendix.

HFx treatment also uses convection and diffusion simultaneously for toxin removal but does not use replacement fluid, in contrast to other blood purification systems. Therefore, to maintain a constant body fluid volume as in the other therapies, the net UF should be zero by controlling the UF and backfiltration. In this HFx model, we set the mean pressure at the dialyser inlet and outlet of the dialysate circuit equal to those in the blood circuit to assign a zero net UF. The procedure to compute the UF rate is presented in Eqs. (A12)-(A15) in the Appendix. Convective toxin clearance for the HFx treatment is also calculated in Eq. (A11) in the Appendix using the UF rate.

### *In vivo *test for model validation

*In vivo *experiments were performed to validate the present computational model. First, we measured urea removal during blood purification in the dialysis unit of Kang's Clinic in Seoul, Republic of Korea, during ten hemodialysis sessions using the AK95 roller pump. Detailed conditions of the clinical measurements are presented in Table 3. Then, we performed model simulations under conditions almost identical to the clinical settings with respect to patient body weight, diffusive mass transfer coefficient of dialyser for urea (KoA_u_), UF rate and blood and dialysate flow rates. The computed results of urea removal were then compared with the *in vivo *clinical data.

**Table 3:** Patient conditions and system settings for *in vivo*dialysis experiments

Parameters	Value	Dimension
Number of patients	18	
Patient body weight	73 ± 9	kg
Predialysis urea concentration	105 ± 12	mg/dL
KoA_u_*	967	mL/min
Blood flow rate	300	mL/min
Dialysate flow rate	500	mL/min
UF rate	0.5	L/h
Treatment time	4	h

### Simulation models

The calculated convective toxin clearance was applied to the proposed mathematical model. Three blood purification therapies (*i.e.*, HF, HDF and HFx) were simulated for both pulsatile and non-pulsatile systems according to the blood flow rate. Then, we compared the total performances of urea and B2M for the pulsatile system with those for the non-pulsatile system.

Next, we performed long-term simulations to compare the delivered treatment dose of the pulsatile system with that of the non-pulsatile system. A treatment schedule of three times per week, 4 h per treatment, was applied to each system. The EKRc and the standard (std) *Kt/V *indices were used to quantify the treatment dose. Here, the EKRc and std *Kt/V *reflect the time-averaged concentration (TAC) and mean pre-treatment concentration (MPC), respectively [7-9]. According to the results of previous studies [9], the required minimum values of EKRc and std *Kt/V *for an anuric patient with a total body water volume of 40 L are 11 mL/min and 2, respectively. The indices are calculated using the time-varying urea concentration profiles in the plasma compartment during 5 weeks of simulation. The indices are defined in the Appendix.

## Results

### *Parameter estimation *and *in vivo *clinical test

**Figure 2:**
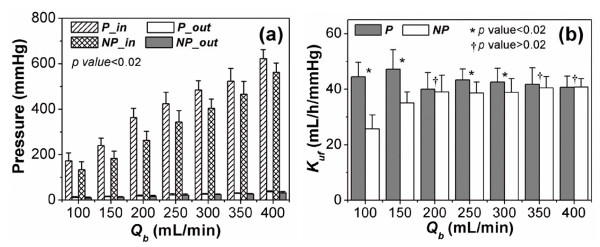
**Variations in dialyser pressure and UF coefficients**. Variation in (a) the pressure at the dialyser inlet and outlet (*p *< 0.02 for differences between *P_in *and *NP_in*) and (b) the UF coefficient of the dialyser (*p *< 0.02 for differences between *P *and *NP*) for *Q_b _*= 100, 150, 250, and 300 according to pump type and the mean blood flow rate. *P_in *and *P_out *are the pressures at the dialyser inlet and outlet, respectively, in the pulsatile pump group. *NP_in *and *NP_out *are the pressures at the dialyser inlet and outlet in the non-pulsatile pump group. *K_uf _*indicates the UF coefficient of the dialyser, *P *and *NP *are the UF coefficients in the pulsatile and non-pulsatile groups, respectively, and *Q_b _*indicates the mean blood flow rate.

The previous *in vitro *experimental result was used to determine the parameters of the computational model, such as the inlet and outlet pressures of the blood circuits, and the UF coefficient of the dialyser according to the blood pumping rate and pump type. Figure 2(a) shows the pressures at the dialyser inlet and outlet of the blood circuit according to the blood flow rate and pump type. Under the same mean flow rate, the dialyser inlet pressure of the pulsatile pump was higher than that of the non-pulsatile pump, whilst the dialyser outlet pressure was almost identical for both pulsatile and non-pulsatile pumps. Figure 2(b) shows the derived UF coefficient according to the blood flow rate and pump type. The UF coefficient of the pulsatile pump was higher than that of the non-pulsatile pump, and the difference in the UF coefficient between the two pumps decreased with increasing blood flow rate.

**Figure 3:**
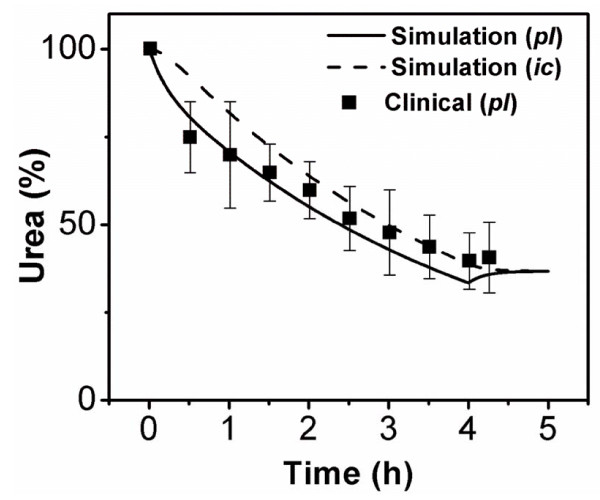
**Comparison of the simulated results with the *in vivo *experiment**. Percent changes of the intracellular urea concentration (------) and plasma urea concentration (----) in the simulation and *in vivo *experiment (■).

As described in the Introduction, urea removal during blood purification was monitored *in vivo *for short treatment times. We compared the measured data with computational results. As shown in Figure 3, the simulated plasma urea concentration profile was very similar to the clinical results; both showed a concentration rebound phenomenon after 4 h of treatment.

### Toxin removal dynamics

Here, HF, HDF and HFx were simulated according to the pump type and blood flow rate. Then, we assessed the effects of the pump type on the toxin clearance performances for urea and B2M.

**Figure 4:**
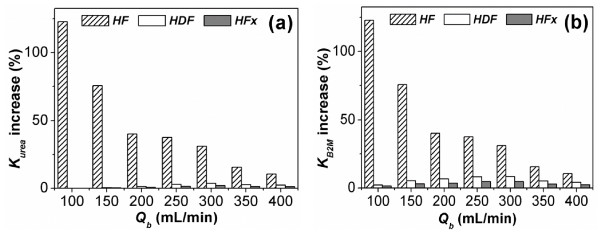
**Percent increase of dialyser clearance by pulsatile pumping**. Difference in dialyser clearance between the pulsatile pump group and non-pulsatile pump group according to treatment type and mean blood flow rate. (a) Percent increase in urea clearance. (b) Percent increase in B2M clearance. *K_urea _*and *K_B2M _*are the dialyser clearances for urea and B2M, respectively, and *Q_b _*indicates the mean blood flow rate.

Figure 4(a) shows the percentage increase in computed urea clearance of the pulsatile pump relative to that of the non-pulsatile pump according to the blood flow rate and therapy. The increase in toxin clearance was highest in the HF treatment followed by the HDF treatment over the entire range of flow rates computed (100-400 mL/min). In the HF treatment, pulsatile pumping increased the urea clearance by a maximum of 122.7% at a blood flow rate of 100 mL/min and a minimum of 10.5% at a blood flow rate of 400 mL/min. The percentage increase in urea clearance was reduced with increases in the blood flow rate in HF. In HDF, urea clearance showed a maximum increase of 3.6% at a blood flow rate of 300 mL/min and a minimal increase of 0.05% at a blood flow rate of 100 mL/min. In HFx, urea clearance showed maximum and minimum increases of 1.9% and 0.03% at blood flow rates of 300 mL/min and 100 mL/min, respectively.

Figure 4(b) shows the percent increase in B2M clearance by pulsatile pumping for the three types of therapy. The HF treatment showed the largest clearance increment followed by the HDF treatment over the entire range of flow rates examined, similar to the case of urea clearance. In HF, the percent increase of B2M clearance was identical to that of urea clearance throughout the entire flow rate. In HDF, the B2M clearance increased by a maximum of 8.3% at a flow rate of 300 mL/min and a minimum of 2.2% at a flow rate of 100 mL/min. In HFx, the B2M clearance showed maximum and minimum increases of 4.7% and 1.5% at flow rates of 300 mL/min and 100 mL/min, respectively. In the cases of the HDF and HFx treatments, the increase in B2M clearance by pulsatile pumping was larger than that of urea clearance.

### Comparison of treatment doses

**Figure 5:**
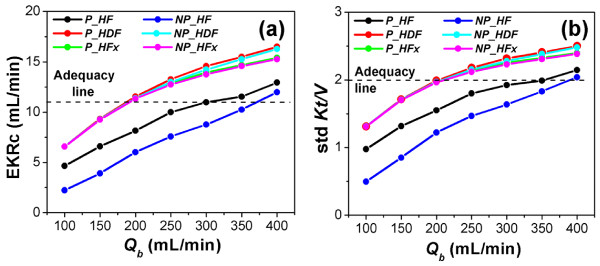
**Comparison of treatment doses; EKRc and std *Kt/V***. This shows the variations of (a) EKRc and (b) std *Kt/V *according to the mean blood flow rate under various treatment conditions and with various pump types. *Q_b _*indicates the mean blood flow rate, *P_HF, P_HDF *and *P_HFx *are pulsatile HF, HDF and HFx, respectively, and *NP_HF, NP_HDF *and *NP_HFx *are non-pulsatile HF, HDF and HFx, respectively.

Figure 5 shows the computed EKRc and std *Kt/V *indices for 12-h weekly treatments in the three types of therapy over the entire range of flow rates examined (100-400 mL/min). For the HF treatment, EKRc and std *Kt/V *were on average 28% and 23% higher in the pulsatile group than in the non-pulsatile group, respectively. However, the superiority of the pulsatile pump was not remarkable in the HDF or HFx treatments, with increments of <2% in both treatments.

To satisfy the adequacy line of EKRc ≥ 11.0 and std *Kt/V *≥ 2.0, the range of the adequate blood flow rate was >200 mL/min for the HDF and HFx treatments under both pulsatile and non-pulsatile conditions. However, for the HF treatment, the ranges of blood flow rate to satisfy the adequacy line of EKRc ≥ 11.0 were >300 mL/min and 400 mL/min under pulsatile and non-pulsatile conditions, respectively. In addition, the ranges of the blood flow rate for the HF treatment to guarantee the adequacy line of std *Kt/V *≥ 2.0 were 350 mL/min and 400 mL/min under pulsatile and non-pulsatile conditions, respectively.

## Discussion

In our previous *in vitro *study, we demonstrated the superiority of a pulsatile pump relative to a non-pulsatile or weak pulsatile pump in terms of the TMP and UF coefficients of the dialyser [3]. Compared to the non-pulsatile pump, the pulsatile pump induced a higher dialyser pressure, such as dialyser inlet pressure and TMP, and a higher UF coefficient at the same mean blood flow rate. The dialyser inlet pressure is influenced by the flow impedance. Therefore, it is clear that the flow wave-form through the dialyser produced by the pulsatile pump induced higher flow impedance than did that produced by the roller pump. As both blood pumps are flow generators and not pressure generators, the pump inducing the higher impedance develops a higher fluid power to maintain the target flow rate, and consequently produces a higher dialyser inlet pressure, which in turn results in a higher TMPm and UF rate. In addition, an instantaneous high pressure developed as the pulsatile pump increased the UF coefficient due to the reduction of membrane layering. However, the difference in the UF coefficient between pulsatile and roller pumps decreased as the pumping rate increased. This implies that an effective pumping frequency exists for reducing membrane layering. In other words, a greater pumping frequency makes the system less efficient in terms of the UF coefficient. These results were limited to basic *in vitro *experiments to determine the UF efficiency of the dialyser device itself.

In this study, theoretical evaluations of the apparatus and its application in patients were performed to assess the effects of pulsatile flow on the efficiency of convective toxin removal in blood purification systems. For this purpose, we devised a new mathematical model, integrating the mass transfer model for the human body [6] with that for the dialyser (Figure 1). For verification of the model, we simulated conventional hemodialysis therapy for 4 h, and the computed urea concentration profile inside the body was compared with clinical observations (Figure 3). Using the verified model, we theoretically predicted the effect of pulsatile pumping on convective toxin clearance (Figure 4) and delivered treatment dose to patients with CRF (Figure 5) for various blood purification therapies, *i.e.*, HF, HDF and HFx.

HF treatment provides only convective toxin removal by UF and injection of replacement fluid, whilst the HDF and HFx treatments provide diffusive toxin removal driven by concentration differences between the blood and dialysate as well as convective toxin removal. The convective toxin removal is dependent on the UF rate and sieving coefficient. The *in vitro *experiment demonstrated that pulsatile pumping improved the UF rate by increasing the UF coefficient and the TMP (Figure 2). The improved UF rate increased the convective toxin removal. Due to the effect of diffusive clearance, the contributions of convective toxin removal to the total clearance in the HDF and HFx treatments were lower than that in the HF treatment. This explains why the increased clearance by pulsatile pumping showed less benefit in the HDF and HFx treatments than in the HF treatment as shown in Figure 4.

The sieving coefficient of the dialyser for B2M was 0.8, whereas the value for urea was 1. These observations indicated that in the HF treatment, B2M removal was 20% less than that of urea under the same UF rate conditions. Thus, the convective clearance for B2M was 20% less than that for urea in HF during which convective toxin clearance occurred. However, HDF and HFx use the mechanisms of diffusive and convective toxin removal simultaneously. In these cases, B2M removal was 20% less than that of urea by the convective effect under the same UF rate conditions. In contrast, the diffusive mass transfer coefficient of the dialyser for B2M [*KoA*_*B2M *_in Eq. (A8) in the Appendix] was 70% less than that for urea, which exceeded the 20% decrease compared to urea by the convective effect. Therefore, in the case of HDF and HFx, the relative portion of convective toxin removal to diffusive toxin removal for B2M was more remarkable than that for urea. These observations explain why the percentage increase in total clearance by pulsatile pumping for B2M was more remarkable than that for urea, as shown in Figure 4.

To test the long-term performance of the three blood purification therapies and predict the effects of pump type on their performance, we simulated the therapies for 5 weeks and obtained the EKRc and std *Kt/V *indices for each case (Figure 5). In terms of the EKRc and std *Kt/V *indices, HDF therapy showed the best performance, followed by HFx therapy. HF therapy showed the poorest performance because it provided only convective toxin removal, whilst the others provided convective and diffusive toxin removals using the dialysis solution. However, the pulsatile effect on the delivered dose was best in HF therapy, with neither HDF nor HFx showing a remarkable effect. As shown in Figure 5, the increases in delivered treatment doses of about 25% for both EKRc and std *Kt/V *in HF therapy suggested that use of a pulsatile pump may become a practical alternative to the conventional method with a non-pulsatile pump for HF treatment.

Although we developed a mathematical model of a CRF patient to assess several blood purification methods, this model is not limited to chronic conditions. It can be used to model an acute renal failure patient by modifying the mass transfer coefficients and considering residual renal function.

Several animal studies have shown that a blood purification system using a pulsatile blood pump can effectively maintain a physiologically stable hemodynamic state, *i.e.*, the heart rate, arterial blood pressure, and hematocrit, and is a plausible alternative to systems using a conventional roller pump [10-12]. Furthermore, our *in vitro *experiment showed that the hematocrit was maintained within the normal range during pulsatile pumping. This means that no blood damage occurred due to the pulse peak pressure during pulsatile blood purification therapy.

The use of a pulsatile blood pump in a blood purification system with a semipermeable membrane was suggested to increase convective toxin removal, which could reduce both treatment time and associated costs.

Although we provided simulated results for the test of the effects of pulsatile pump on toxin removal, there are some limitations to our study. First, we have validated our numerical patient model by comparing the simulated results with that the clinical test only using a conventional non-pulsatile system. However, a comparison with the experiment using pulsatile pump was not attempted because of no available pulsatile pump that can be applied to patients among commercialized blood pumps. In addition, the input parameters of the model, such as the pressures at the dialyser inlet and outlet and UF coefficients, were estimated from *in vitro *rather than *in vivo *experiment.

## Conclusions

We proposed a new mathematical model to assess the toxin removal efficiency of blood purification systems in patients, integrating the mass transfer model for a human body with a dialyser. *In vivo *experiments were also performed to verify clinically the model results, respectively. Using the verified model, we simulated the effect of pulsatile pumping on convective toxin clearance and delivered treatment dose to patients with CRF for various blood purification therapies. Compared with non-pusatile pumping method, the increases of urea and B2M clearances by pulsatile pump were highest in HF treatment (122.7% and 122.7%, respectively), followed by HDF (3.6% and 8.3%, respectively) and HFx (1.9% and 4.7%, respectively). EKRc and std *Kt/V *were on average 28% and 23% higher, respectively, in the pulsatile group than in the non-pulsatile group in HF treatment.

The superiority of pulsatile pumping is significant in HF treatment, which relies only on convection for toxin removal. In both HDF and HFx treatments, the effectiveness of pulsatile pumping on B2M removal, which depends more on convection compared to urea removal, was more remarkable than that on urea removal. Finally, pulsatile system will help any blood purification treatments, whose mechanism of toxin removal is mainly convection, get better performance than conventional non-pulsatile system.

## Appendix

### A1. *In vitro *experiment for identification of model parameters

**Figure 6:**
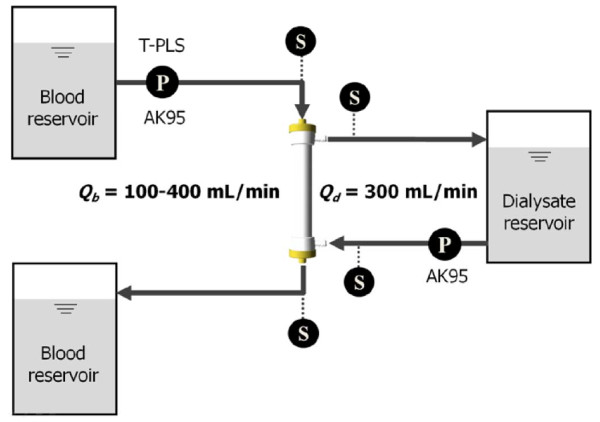
**Schematic diagram of the *in vitro *experiment with the dialysis circuit**. There are two blood reservoirs, one dialysate reservoir, one dialyzer, two blood pump, and four pressure sensor. P indicates the blood pump; P in the blood circuit can be the T-PLS pulsatile pump or the AK95 roller pump, and P in the dialysate circuit is the AK95 roller pump. S indicates a pressure sensor.

**Figure 7:**
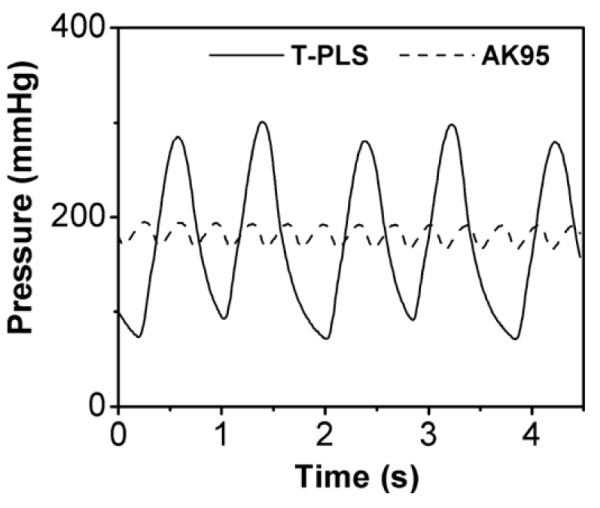
**Pressure wave forms at the dialyser inlet of the blood circuit**. This is measured data from the *in vitro*experiment using bovine whole blood as working fluid. Here, (-----) represents the pressure wave form produced by the T-PLS pulsatile pump and (-------) by the AK95 roller pump.

A schematic of the experiment is shown in Figure 6. The experiments were performed for a pulsatile pump (T-PLS; BHK Inc., Seoul, Republic of Korea) and a non-pulsatile pump (AK95 roller pump; Gambro Inc., Hechingen, Germany) under various flow rate conditions from 100 to 400 mL/min in increments of 50 mL/min. Figure 7 shows the pressure waveforms at the dialyser inlet generated by the two pumps. The AK95 pump generated a very weak pulsatile flow that can be considered as almost non-pulsatile flow. Each test was performed using bovine whole blood (heparin of 16 000 IU/L, Hct = 31%, T = 37°C). In the dialysate circuit, we used the AK95 roller pump at a constant flow rate of 300 mL/min for all test cases. A polysulphone dialyser (FX60; FMC Inc., Frankfurt, Germany) was used with normal saline as the dialysis solution. An ultrasonic flow sensor (T109; Transonic Systems Inc., Ithaca, NY) was placed in the blood circuit before the dialyser to measure the flow rate. Four pressure sensors (Pressure transducer; Sensys Inc., Seoul, Republic of Korea) were inserted before and after the dialyser in the blood and dialysate circuits. In the experiment, according to the pump type and flow rate, we measured the four pressures (at the dialyser inlet and outlet of the blood circuit, and at the dialyser inlet and outlet of the dialysate circuit) and UF coefficient of the dialyser. Before starting the test, we recirculated bovine blood through the dialyser at 200 mL/min for 2 h to develop a sufficient protein layer as suggested in a previous study [13].

The goal of the *in vitro *experiment was to establish reference data for the variation in TMPm and UF coefficient according to pump type and flow rate. Thus, the parameters such as TMPm and the UF coefficient for a specific condition of flow rate and pump type can be obtained by interpolating the reference data.

### A2. Governing equations of the mathematical model

The time derivatives of the intracellular fluid (*V*_*ic*_), interstitial fluid (*V*_*is*_) and plasma (*V*_*pl*_) can be expressed as(A1)(A2)(A3)

where *k*_*f *_is the osmotic filtration coefficient at the cellular membrane, *O*_*ic *_and *O*_*is *_are the osmotic concentrations of the intracellular and interstitial compartments, respectively, *F*_*a *_is fluid filtration rate at the arterial capillaries, *R*_*v *_is the fluid reabsorption rate at the venous capillaries and *Q*_*f *_is the UF rate.

The time derivatives of the solute concentrations in the intracellular (*M*_*s,ic*_) and extracellular (*M*_*s,ex*_) compartments are expressed as follows:(A4)(A5)

where *η*_*s *_is the mass transfer coefficient of solute *s *at the cellular membrane, *β*_*s *_is the equilibrium ratio of solute *s*, *C*_*s,ic *_and *C*_*s,ex *_are the intracellular and extracellular concentrations of solute *s*, respectively, *G*_*s *_is the generation rate of solute *s *and *J*_*s *_is the solute transfer rate through the dialyser membrane. We assumed *G*_*urea *_to be 6.24 mg/min [2], and *J*_*s *_is derived below:(A6)

where *α*_*s *_is the Gibbs-Donnan equilibrium ratio of solute *s*, *r *is the plasma water fraction and *K*_*s *_is the clearance of solute *s. *HDF or HFx treatments use simultaneous diffusion and convection for toxin removal. Thus, toxin clearance is calculated as the sum of diffusive clearance (*Kd*_*s*_) and convective clearance (*Kc*_*s*_) as follows:(A7)

The diffusive clearance is expressed as(A8)

where *KoA*_*s *_is the diffusive mass transfer coefficient of the dialyser for solute *s *and *Q*_*b *_and *Q*_*d *_are the blood and dialysate flow rates through the dialyser, respectively. The convective clearance is expressed as(A9)

where *Si*_*s *_is the membrane sieving coefficient of solute *s. T*, termed the transmittance, represents the mL/min increase in clearance for each mL/min of filtration and can be calculated as follows:(A10)

The HF treatment uses only convection for toxin removal, and thus the convective clearance is simply calculated as follows:(A11)

More detailed equations were described previously by Ursino *et al*. [6] and Depner and Garred [2].

### A3. Derivation of the internal UF rate for the HFx treatment

To calculate the internal UF rate in the HFx treatment model, we introduce the UF coefficient per unit length, which is determined by dividing the UF coefficient by the dialyser length as follows:(A12)

where *K*_*ufl *_is the UF coefficient per unit length and *L *is the dialyser length. As a simplified approximation, the local pressure distributions along the dialyser fibres in the blood and dialysate circuits are described as follows:(A13)(A14)

where *x *indicates the local position along the dialyser, *P*_*b*_(*x*) and *P*_*d*_(*x*) are the mean pressures at local position *x *in the blood circuit and dialysate circuit, respectively. The UF rate, which is the primary determinant of the convective clearance of the toxin, is calculated as follows:(A15)

where *M *is a local point where neither UF nor backfiltration is generated. The internal UF rate is directly related to the convective toxin clearance in the HFx treatment, and therefore, the convective clearance for the HFx treatment is calculated using Eq. (A9) in the Appendix.

### A4. Definition of criterion indices

The time-averaged concentration of solute *s *(*TAC*_*s*_) is calculated by integrating the concentration profile of the solute in the plasma compartments with respect to time as follows:(A16)

Equivalent renal clearance (*EKR*_*s*_) is calculated as follows:(A17)

*EKRc*_*s *_is the corrected value of *EKR*_*s *_for the normalised water volume, 40 L, as follows:(A18)

where *V *is the total body fluid.

The mean pre-treatment concentration of solute *s *(*MPC*_*s*_) is acquired by averaging the pre-treatment concentrations in the plasma compartment at steady state, and the weekly std *Kt/V *is then calculated as follows:(A19)

where *t *is the total time. All equations are adapted from Ursino *et al*. [6] and Depner and Garred [2].

## Competing interests

The authors declare that they have no competing interests.

## Authors' contributions

KML carried out all the computations and analyzed the computed data. EBS designed the computations, analyzed and interpreted the computed data. All authors were actively involved in the writing of the manuscript, read it and approved the final manuscript.

## Authors' information

KML received Ph.D. from Dept of Biomedical Engineering of Seoul National University, South Korea at 2008 and thereafter worked for the Biosystems Engineering Lab of Kangwon National University, South Korea, as a post doc. And now he is working for the department of Biomedical Engineering of Johns Hopkins Univ. as post doc.

EBS finished undergraduate course at Seoul National University and received Ph.D. from the Mechanical Engineering Dept. of KAIST (Korea Advanced Institute of Science and Technology) at 1994. He received another Ph.D. at the Dept. of Physiology (Medical School) of Kyoto University, Japan, at 2008. He became a full professor of Kangwon National University from 2004.
